# Quality of life of Croatian veterans’ wives and veterans with posttraumatic stress disorder

**DOI:** 10.1186/s12955-014-0136-x

**Published:** 2014-09-11

**Authors:** Tina Peraica, Anđelko Vidović, Zrnka Kovačić Petrović, Dragica Kozarić-Kovačić

**Affiliations:** Department of Psychiatry, Referral Centre for the Stress Related Disorders of the Ministry of Health of Croatia, University Hospital Dubrava, Avenija Gojka Šuška 6, Zagreb, Croatia; Department of Psychopharmacology, Croatian Institute for Brain Research, School of Medicine, University of Zagreb, Šalata 12, Zagreb, Croatia

**Keywords:** Combat PTSD, War veterans, Veterans’ wives, Quality of life, WHOQOL-BREF

## Abstract

**Background:**

Posttraumatic stress disorder (PTSD) has a great impact on a patient’s quality of life (QoL). People in long-term contact with a person suffering from PTSD, such as family members, may also consequently develop various emotional problems.

**Methods:**

We investigated whether chronic combat PTSD is related to lower QoL of veterans’ wives. The study included 164 veterans with PTSD, 281 PTSD veterans’ wives, 115 healthy non-veterans’ wives and 223 men without PTSD. All participants were assessed for psychiatric morbidity (Mini International Neuropsychiatric Interview) and measures of QoL (World Health Organization Quality of Life Questionnaire – short form). In veterans, the symptoms were evaluated using Clinician Administered PTSD Scale (CAPS).

**Results:**

The pattern of differences between the study groups was similar across all QoL domains. Men without PTSD had the highest scores, followed by women who were not married to PTSD patients (significantly different across all four QoL domains). PTSD veterans’ wives tended to had lower scores than either of these groups. Significant differences between PTSD veterans’ wives and women who were not married to PTSD patients were observed in three (out of four) QoL domains: psychological health, Z = 2.907, P = 0.022; social relationships, Z = 3.285, P = 0.006; and environmental domain, Z = 5.317, P < 0.001. The lowest scores were recorded in PTSD veterans (significantly lower than PTSD veterans’ wives in every domain except environmental). The wives who reported to be ill at the time tended to have lower scores across all four domains (P < 0.05) (low to moderate correlation; physical health, *ρ* = −0.56; psychological health, *ρ* = −0.42; social relationships, *ρ* = −0.30; environmental, *ρ* = −0.33), as well as those who sometimes in their lives they sought professional help due to having problems with their husband (P < 0.05) (very low to low correlation; physical health, *ρ* = −0.37; psychological health, *ρ* = −0.38; social relationships, *ρ* = −0.30; environmental, *ρ* = −0.15).

**Conclusion:**

Our results indicate the negative impact of husband’s chronic PTSD on overall QoL, psychological health, social relations, and environmental area of QoL of veteran’s wives. Assessment of QoL may be important during the intervention and planning of specific treatment and rehabilitation programs for the families of war veterans.

## Background

SINCE mental disorders can have pervasive effects on all areas of a person’s life (physical, psychological, social, occupational, and so on), there is a need to investigate their impact on a patient quality of life (QoL) and integrate the QoL approach into clinical practice [1]. In general population, anxiety disorders show the highest prevalence, ranging between 15% and 20% [2], whereas the prevalence of posttraumatic stress-disorder (PTSD) ranges between 1% and 14% [3]. Many authors explored the effect of anxiety disorders on QoL [4-6] and found that these disorders have a substantial negative impact on patient functioning in many areas and reduce patient QoL [5-7].

QoL-related data in specific types of anxiety disorders and especially in patients with PTSD are scarce [5,8]. Among different subtypes of anxiety disorders, panic disorder and posttraumatic stress disorder (PTSD) have the greatest impact on patient QoL [9]. Findings suggest that patients with PTSD have reduced QoL [10,11], including self-reported decreased well-being, poor physical health, and marital and occupational difficulties [12-14]. Additionally, war veterans with PTSD report less intimacy, less self-disclosure and expressiveness, and more hostility and physical violence toward their marital partners than war veterans without PTSD [15]. All these difficulties in marital and family functioning have a sustained negative effect on veterans and their families [16-19]. In families of war veterans, domestic violence is often present and divorce rates are high [20], both indicating detrimental effects of PTSD on overall family functioning and the QoL of the family members. Members of the families of war veterans with PTSD are often called the “hidden victims” of war trauma [21]. Previous research showed that close and long-term contact with a person with PTSD may cause chronic stress and various emotional problems, including higher levels of depressive symptoms and anxiety, concentration problems, emotional exhaustion, pain syndromes, and sleeping problems in persons providing help [17,18].

Different studies or organizations have proposed different definitions of QoL [1]. A World Health Organization Quality of Life Questionnaire – short form, WHOQOL-BREF, which captures many of a person’s own views of their well-being, was designed by the WHO Quality of Life Group [22,23]. It offers the most optimal approach to cross-cultural comparison of QoL and it is available in many languages [23].

Although PTSD causes difficulties in many aspects of life, previous studies have evaluated only the influence of PTSD on family functioning in war veterans and civilian victims [15,18] and their family members [24], focusing on the secondary traumatization [16,17]. To our best knowledge, the impact of long-term and chronic PTSD in war veterans on the overall and specific aspects of QoL in their wives has not been investigated nor mutually compared. There is only one naturalistic study that assessed the psychological effects of a lifestyle management course on war veterans and their spouses [25].

Since chronic PTSD is known to have detrimental impact on war veterans’ QoL, we tested the hypothesis that wives of veterans with PTSD also have impaired QoL.

## Methods

### Participants

A total of 783 subjects were recruited including 164 male PTSD veterans, 281 PTSD veterans’ wives, 115 healthy non-veteran women, and 223 men without PTSD.

War veterans with PTSD were recruited during the intensive treatment program at the University Hospital Dubrava, Department of Psychiatry, Referral Centre for the Stress Related Disorders of the Ministry of Health of Croatia, in Zagreb, Croatia. They participated in the 1991–1995 war in Croatia and the mean duration of their active military service was 38 ± 21 months. Four of them had a detention camp experience. At the time of the assessment, they had been in treatment for a mean period of 7 ± 5 years.

The group of PTSD veterans’ wives was recruited during a medical checkup at the Clinic for Tumors in Zagreb, Croatia, within a comprehensive health assessment organized by the Ministry of War Veterans of Croatia. The assessment included psychiatric evaluation in addition to thorough physical examination. These women did not actively participate in combat activities and did not have any civilian war trauma or earlier traumatic experiences. Their husbands’ PTSD diagnosis was assessed by a psychiatrist and confirmed in an expert examination for compensation seeking purposes. At the time of the assessment, their husbands had been in treatment for PTSD for 10 ± 4 years. Sixty seven women were excluded because of their active combat participation or civilian war experience or earlier psychological traumatizations.

Women in the comparison group were recruited from among the women undergoing a regular systematic physical examination at the University Hospital Dubrava in Zagreb. The inclusion criterion was that their husbands did not have any combat experience and were not treated for PTSD or any other mental disorder. Eleven women were excluded because their husbands were treated for mental disorders.

The fourth group consisted of healthy men undergoing a regular physical examination at the University Hospital Dubrava in Zagreb. A major number of them (n = 160, 71.7%) had a war-combat experience. Also, 5 men in this group were excluded because they met the PTSD criteria.

### Measures

Croatian version 5.0.0 of Mini International Neuropsychiatric Interview (M.I.N.I.) was used to evaluate psychiatric morbidity in all study subjects [26]. All veterans with PTSD met the diagnostic criteria for PTSD based on the Diagnostic and Statistical Manual of Mental Disorders, 4th edition – text revision (DSM-IV-TR) [3]. Additionally, the diagnosis was confirmed by using the Clinician Administered PTSD Scale (CAPS) [27]. Diagnostic groups were formed as shown in Table 1.

**Table 1:** **Participants’ characteristics and comparison of scores across QoL domains**

	**PTSD veterans (** ***N*** **= 164)**		**PTSD veterans’ wives (N = 281)**		**Women (N = 115)**		**Men without PTSD (N = 223)**		**P**
Age; median (Q1-Q3)	44 (40–48)		48 (42–53)		45 (33–56)		39 (35–44)		<0.001
Education; N (%)									<0.001
None	0	(0.0)	0	(0.0)	0	(0.0)	3	(1.3)	
Primary	12	(7.3)	46	(16.4)	9	(7.8)	6	(2.7)	
Secondary	135	(82.3)	184	(65.5)	80	(69.6)	151	(67.7)	
Tertiary	17	(10.4)	51	(18.1)	26	(22.6)	63	(28.3)	
Marital status; N (%)									<0.001
Single	21	(12.8)	0	(0.0)	21	(18.3)	44	(19.7)	
Married	130	(79.3)	273	(97.2)	73	(63.5)	169	(75.8)	
Living as married	4	(2.4)	2	(0.7)	4	(3.5)	4	(1.8)	
Separated	8	(4.9)	0	(0.0)	2	(1.7)	5	(2.2)	
Divorced	1	(0.6)	3	(1.1)	10	(8.7)	1	(0.4)	
Widowed	0	(0.0)	3	(1.1)	5	(4.3)	0	(0.0)	
Primary/Comorbid diagnosis; N (%)									<0.001
None	24	(14.6)	254	(90.4)	104	(90.4)	221	(99.1)	
Affective disorders	47	(28.7)	21	(7.5)	5	(4.3)	0	(0.0)	
Anxiety disorders	58	(35.4)	5	(1.8)	6	(5.2)	2	(0.9)	
Psychotic disorders	32	(19.5)	1	(0.4)	0	(0.0)	0	(0.0)	
Personality disorders	3	(1.8)	0	(0.0)	0	(0.0)	0	(0.0)	
Combat experience; N (%)	164	(100.0)	0	(0.0)	0	(0.0)	160	(71.7)	<0.001
Currently ill; N (%)	140	(85.4)	90	(32.3)	28	(24.3)	6	(2.7)	<0.001
WHOQOL-BREF scores; median (Q1-Q3)									
Overall QoL	3 (2–3)		3 (3–4)		4 (3–4)		4 (4–4)		<0.001
General health	2 (1–3)		3 (3–4)		4 (3–4)		4 (4–5)		<0.001
Physical health	38 (25–50)		63 (44–75)		69 (56–81)		88 (81–94)		<0.001
Psychological health	38 (25–50)		63 (50–69)		69 (56–81)		81 (75–94)		<0.001
Social	50 (31–63)		69 (50–75)		75 (56–81)		81 (75–94)		<0.001
Environmental	56 (44–69)		56 (44–63)		69 (56–75)		75 (69–88)		<0.001

The differences in categorical variables were tested with Pearson’s *χ* ^*2*^ tests and continuous variables were compared by using Kruskal–Wallis one-way analysis of variance by ranks. Abbreviations: QoL, quality of life; Q1–Q3, interquartile range; WHOQOL-BREF, The World Health Organization Quality of Life Questionnaire – short form; PTSD, posttraumatic stress disorder.

Clinical interviews were performed by experienced licensed psychiatrists and psychologists with expertise in the field of psychotrauma.

After the structured interviews, the participants were given WHOQOL-BREF [28,29]. At least one of the authors of this study was present to provide help to the participants while they were filling out the questionnaire. We compared QoL in four groups of participants, i.e., male PTSD veterans, PTSD veterans’ wives, women and men without PTSD.

The study was conducted from January to December 2008 in parallel at both institutions. The chances for an individual to be enrolled in this study were the same for all groups according to the inclusion criteria. The participants lived in urban areas in the same region, which is important because previous research in QoL in Croatia showed that inequalities in regional development levels substantially influenced the QoL of the population [30]. All participants gave their written informed consent before taking the structured clinical interview. The study was approved by the Ethics Committees of the University Hospital Dubrava and Clinic for Tumors in Zagreb.

### Instrument

WHOQOL-BREF is the most frequently used questionnaire for QoL evaluation [28]. It consists of 24 items, which measure the four main domains of the QoL: a) physical health (7 items; activities of daily living, dependence on medical substances and medical aids, energy and fatigue, mobility, pain and discomfort, sleep and rest, work capacity), b) psychological health (6 items; bodily image and appearance, negative feelings, positive feelings, self-esteem, spirituality/religion/personal beliefs, thinking, learning, memory and concentration), c) social relationships (3 items; personal relationships, social support, sexual activity), and d) environment (8 items; financial resources, freedom, physical safety and security, accessibility and quality of health care, home environment, opportunities for acquiring new information and skills, participation in and opportunities for recreation/leisure activities, physical environment). It also has two general items that separately measure the person’s overall perception of QoL (overall QoL) and general perception of health (general health). The level of satisfaction or degree of agreement for each item is rated on a 5-point Likert scale. The score for each domain is defined as the sum of individual item scores on subscales transformed into a scale from 0 to 100.

### Statistical analysis

The normality of continuous variables was tested per group using Shapiro-Wilk’s test. Variables pertaining to the four QoL domains did not follow a Gaussian distribution even after every commonly used transformation. Due to the significant deviation from normality, group differences and associations between variables were analyzed with non-parametric tests. Differences between groups in two general items and four domains of QoL were tested with Kruskal–Wallis one-way analysis of variance by ranks. Every test was followed by multiple comparisons of mean ranks between pairs of groups (Mann–Whitney tests) and the resulting p-values were corrected by Bonferroni method.

Associations between variables were tested with Spearman’s rank correlation. Age, overall QoL, general health, four QoL domains, length of active duty, treatment duration (PTSD veterans and husbands of PTSD veterans’ wives, respectively) were analyzed as continuous variables, whereas education was used as an ordinal variable. Marital status and primary/comorbid diagnostic group variables were dichotomized (married vs. others; disorder vs. no disorder) and coded with 0 and 1. Other variables (combat experience, war camp experience, being currently ill, sought professional help) were used as dichotomous variables coded with 0 and 1. We correlated available data for each group with two general items and four domains of QoL. As a result, four sets (one for each group) of correlations were created, with the number of bivariate correlations ranging from 30 to 54 per group (depending on the available variables for particular group). Generated p-values were corrected by the false discovery rate method to minimize the chance of type I error [31] (for every p-value, the corresponding f value was calculated; the effect was significant at the *α* = 0.05 level if p < f).

The strength of the association between variables was interpreted according to Hinkle et al. [32]**:** 0.00 – 0.30, very low; 0.30 – 0.50, low; 0.50 – 0.70, moderate; 0.70-0.90, high; 0.90 – 1.00, very high.

Categorical variables were compared between groups with Pearson’s *χ*^*2*^ tests. The statistical analyses were performed with Statistica 8.0 (StatSoft Inc., Tulsa, OK, USA).

## Results

### Sociodemographic characteristics and psychiatric morbidity of participants

The groups differed in age, with PTSD veterans’ wives being slightly older than other groups (Table 1). The difference between the comparison group of women and PTSD veterans was the only one that did not reach a statistical significance (Z = 0.162, P = 0.990). Although groups differed in education level, majority of participants in each group had completed secondary education (Table 1). The greatest proportion of participants with tertiary education was observed in the group of men without PTSD. PTSD veterans’ wives had similar education level as did women who were not married to PTSD patients (P = 0.070). As for the marital status, most of the PTSD veterans’ wives were married. The group of women who were not married to PTSD patients had diverse marital status (Table 1). Their marital status was different when compared to PTSD veterans (P < 0.001), as well as men without PTSD (P < 0.001).

While majority of PTSD patients had at least one comorbid disorder, the prevalence of psychiatric disorders in other groups was relatively low (Table 1). Only two participants satisfied the criteria for psychiatric disorders among men without PTSD. Disorders were more prevalent in two groups of women, but no difference in psychiatric morbidity was observed between these two groups (P = 0.165). A substantial proportion of men without PTSD experienced combat (Table 1), but none developed psychiatric disorder associated with that experience. The greatest proportion of participants that reported current illness was found in the group of PTSD veterans (Table 1). On the other hand, only a few men without PTSD reported that they were currently ill. No difference was found between the two groups of women (P = 0.119).

### Measures of reliability

For the diagnosis of PTSD and comorbid diagnoses, inter-rater agreement was excellent (k = 0.91). To check the internal consistency reliability, we calculated the following Cronbach’s alpha coefficients: physical health = 0.92, psychological health = 0.91, social relationships = 0.79 and environment = 0.82, and the total for 26 items = 0.96. These coefficients were higher than the ones reported previously by WHOQOL Group for Croatian sample [29].

### Group comparison across QoL domains

**Figure 1:**
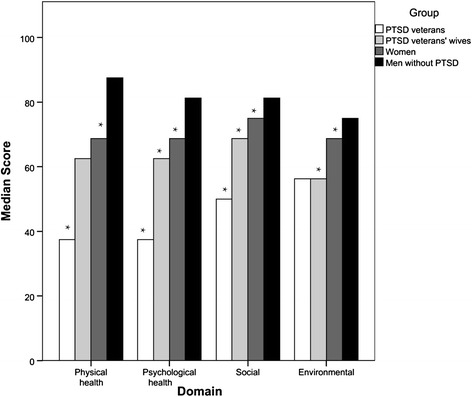
**Comparison of scores across quality of life domains between groups.** Veterans with posttraumatic stress disorder (PTSD), PTSD veterans’ wives, women who were not married to PTSD patients, and men without PTSD (groups are represented by columns in different colors) are being compared across four main Quality of Life domains. Asterisk (*) indicates that particular group differs significantly (P < 0.05) from the first, or every other group to the right in the corresponding domain (i.e. PTSD veterans have significantly lower scores from every other group in all four domains).

A similar pattern of differences between groups was observed in each domain (Figure 1, Table 1). PTSD veterans had the lowest scores across all domains. The scores were significantly lower in comparison with PTSD veterans’ wives in three QoL domains: physical health, Z = 9.040, P < 0.001; psychological health, Z = 8.943, P < 0.001; social relationships, Z = 6.265, P < 0.001. PTSD veterans’ wives had significantly lower scores than women who were not married to PTSD patients in three QoL domains: psychological health, Z = 2.907, P = 0.022; social relationships, Z = 3.285, P = 0.006; environmental domain, Z = 5.317, P < 0.001. Men without PTSD had the highest scores in every domain, significantly higher than women who were not married to PTSD patients. The differences in two general items (overall QoL and general health) followed the pattern similar to QoL domains (Figure 1, Table 1) with PTSD veterans having the lowest and men without PTSD the highest scores. PTSD veterans’ wives had lower overall QoL (Z = 5.10, P < 0.001), as well as general health (Z = 2.73, P = 0.038) scores, in comparison to group of women who were not married to PTSD patients.

### Correlation analyses

Significant bivariate correlations were especially pronounced in the group of PTSD veterans’ wives (Table 2). Age was negatively associated with QoL scores, as opposed to education, which correlated positively with the two general items and each QoL domain. Duration of their husbands’ PTSD treatment negatively correlated only with physical health domain.

**Table 2:** **Correlations between participant characteristics and scores across QoL domains in PTSD veterans’ wives**

			**Domain**			
	**Overall QoL**	**General health**	**Physical health**	**Psychological health**	**Social relationships**	**Environmental**
Age	−0.10	−0.20*	−0.24*	−0.22*	−0.21*	−0.08
Education	0.26*	0.19*	0.19*	0.22*	0.17*	0.20*
Children	−0.02	0.01	−0.03	−0.01	0.04	−0.05
Currently ill	−0.26*	−0.56*	−0.56*	−0.42*	−0.30*	−0.33*
Psychiatric disorder	−0.17*	−0.20*	−0.30*	−0.34*	−0.25*	−0.13*
Husband’s treatment duration	0.07	−0.06	−0.16*	−0.09	−0.03	0.04
Sought professional help	−0.13	−0.27*	−0.37*	−0.38*	−0.30*	−0.15*

The values represent Spearman’s correlation coefficient (*ρ*), N = 281. Asterisk (*) indicates significant correlation (P < 0.05) corrected for multiple correlations by false discovery rate method. Abbreviations: QoL, quality of life; PTSD, posttraumatic stress disorder.

However, all of the above mentioned associations could be regarded as weak, or negligible, as reflected in very low Spearman’s correlation coefficients (<0.30). Women who reported that they were currently ill tended to have lower QoL scores, especially in general health and physical health domain, as reflected by moderate negative correlation (Table 2). Low negative correlation was found between the presence of psychiatric disorder and psychological health domain. The wives who sought professional help due to the problems they had with their husbands (who did not have a psychiatric diagnosis) tended to have lower QoL scores (low negative correlation in physical and psychological health domains; Table 2).

PTSD veterans who reported that they were currently ill tended to have lower general health (*ρ* = −0.36, P < 0.05) and physical health scores (*ρ* = −0.31, P < 0.05).

The similar low correlations were also observed in the group of women who were not married to PTSD patients (general health: *ρ* = −0.37, P < 0.05; physical health: *ρ* = −0.33, P < 0.05). Significant, but weak (very low correlation) associations were found in the comparison group of women. Environmental domain scores positively correlated with age (*ρ* = 0.27, P < 0.05) and education (*ρ* = 0.28, P < 0.05). The presence of psychiatric disorder in this group of women was negatively associated with physical health (*ρ* = −0.26, P < 0.05) and psychological health (*ρ* = −0.24, P < 0.05) scores.

The only significant correlation in the group of men without PTSD was that of age with general health scores (*ρ* = −0.34, P < 0.05).

## Discussion

Our study showed lower overall QoL in veterans with PTSD than in the wives of veterans with PTSD, whereas QoL in both of these groups was lower in comparison with healthy male and female groups. With respect to lower QoL in wives of veterans with PTSD in comparison with the healthy female group, we may assume that the reason was their husbands’ chronic illness, i.e., PTSD. Previous QoL studies among caregivers (mostly mothers) of patients with mental disorders [33] and wives of patients with mental disorders [34] showed they had a significantly lower QoL in comparison with general population. Similar results were obtained in a study among wives of patients with chronic physical diseases [35].

The level of QoL of veterans with PTSD in our study was consistent with the results of previous studies, which showed that patients with PTSD had reduced overall QoL [4,6,36], and difficulties in multiple areas of functioning including family [10,15,24], psychological health [37,38], physical health [13], social [12], and occupational functioning and overall well-being [11,14]. Previous studies investigating the effects of war veterans PTSD on their wives, but not their QoL, showed that these women had an increased risk of developing psychological and relational distress [10,16-19,39]. Although it could be argued that some aspects of secondary traumatization (like emotional problems) may be also considered as measures of QoL, in previous studies specific measurement instruments of QoL were not applied.

National-level studies investigating gender effect on the subjective perception of QoL in general population [40], patients with different mental disorders [41], and patients with PTSD [14] showed that gender had no effect on the QoL self-assessment. Our results may indicate that the mental disorder in their husbands (PTSD) might be directly associated with QoL in the wives of war veterans.

Higher age of wives of veterans with PTSD was weakly associated with their reduced QoL in all QoL areas except environmental (without significant association). The effect of age on subjective QoL reported in previous studies has been inconsistent and a meta-analysis of studies showed that correlation between age and subjective QoL was near zero [42]; investigators have begun to focus not so much on age per se but on life cycle patterns [40].

Previous research showed that education level, higher age at the beginning of active war combat, higher socioeconomic status, and better family relations were protective factors for the development of PTSD [43]. Men in the healthy group in our study had a significantly higher level of education in comparison with veterans with PTSD (tertiary education: 28.3% vs. 10.4%), which could be the reason why they had not developed PTSD despite the fact that 71% of them had war experience. Higher education level was also associated with higher overall level of QoL, general health, and satisfaction in all QoL areas (psychological, physical, social, and environmental) in wives of veterans with PTSD and higher satisfaction with environment in the comparison group of women. The higher education level had positive effects on better QoL and subjective well-being in previous investigations [33,44].

Marital status and partner relationships have been frequently found to have a positive influence on life satisfaction, course of physical and mental disorders, and recovery [45,46]. Our study has not confirmed these findings, since no correlations between marital status and QoL domains was found in any of groups (data not showed). Since the proportion of wives who divorced their husbands with PTSD in our study was lower than in the comparison group and general population in Croatia (around 15%) [47], we may assume that they remained married irrespective of the marital difficulties [39]. The most frequently reported reasons for women to stay married to veterans with PTSD are perceived lack of choice, intensely felt moral obligation, and fear for husband’s life [48].

In patients with PTSD, comorbidity was present in about 80% of the cases [49,50], including mood disorders, other anxiety disorders, somatization, substance abuse, and dissociative disorders and this is generally in line with our findings (Table 1).

We did not investigate the effect of comorbid diagnoses on QoL in veterans with PTSD, but rather focused on the QoL of the wives of veterans with PTSD in whom the prevalence of mental disorders was within the range of European countries [51].

A substantial number of veterans’ wives (N = 98, 34.9%) sought professional psychological help due to the problems they had with their husbands after the war, but none were in psychiatric treatment. It is possible that QoL is a better indicator of the suffering of wives of war veterans with PTSD than mental disturbances alone.

Self-reported current illness (physical and mental) in majority of our study participants, except male comparison group, was found to be an important factor associated with the overall perception of QoL in the wives of veterans with PTSD. It was also associated with the general perception of health and physical health in veterans with PTSD and female comparison group, as shown in a previous study [52].

In our study, overall QoL and QoL in all four specific domains was lower in the wives of veterans with PTSD than in female comparison group and significantly lower in psychological health, social relations, and environment domains. Angermeyer et al. [34] found the difference in the psychological and social domains of QoL between wives of patients with mental disorder and general population, but not in the environmental domain. With respect to the questions that cover the environment domain in our study, we may assume that some other factors, like employment status, income, and living conditions [53], in addition to husband’s disease influenced the low QoL in environment domain. This finding is in line with results from a study investigating objective indicators of QoL in Croatia, which showed that Croatians are less satisfied with environmental factors in comparison with the population in European Union [30], and more often report problems related to financial status, lack of information, availability of medical and social services, and feeling of social exclusion [44]. On the other hand, this was the only QoL domain where no difference was found between wives of war veterans with PTSD and veterans themselves, which may be explained by similarities in their objective living conditions, greater need for health and social care, and low mobility.

The strengths of our study are a large sample and the fact that this is the first study investigating the QoL of wives of veterans with PTSD by using a specific questionnaire, which captures many personal views (e.g. subjective perceptions) of QoL.

There are several limitations of the study. First, we did not explore the impact of somatic comorbidity and health status on QoL. Second, the study design was a cross-sectional (correlational) thus precluding causal inference. A long-term longitudinal assessment of QoL in PTSD patients and their partners in comparison with the control group would be needed to allow for evaluating a causal relationship. Third, multiparametric regression analyses could not be performed because the data did not meet the criteria for parametric tests. Fourth, there was no “objective” (i.e. external) assessment of QoL and psychopathological symptoms were not included in the QoL measuring instrument. Fifth, it remains questionable how valid and comparable are our results with other research findings obtained with different existing instruments for measuring QoL.

## Conclusions

Our study results indicate the negative impact of husband’s chronic PTSD on the overall QoL, psychological health, social relations, and environmental area of QoL of vesteran’s wives.

Today, the focus is on the principles of “promotion of mental health”, which can also be regarded as a means of improving QoL in general population (healthy, at-risk, and those already suffering from mental disorders) [1].

According to our findings, it is necessary to develop specific preventative actions and treatment and rehabilitation programs for the families of war veterans, especially for veteran’s wives. These programs should include interventions such as family group support, psychoeducation, counseling and advisory services.

## Abbreviations

PTSD: Posttraumatic stress disorder
QoL: Quality of life
WHO: World Health Organization
